# Socioeconomic Correlates and Determinants of Cardiorespiratory Fitness in the General Adult Population: a Systematic Review and Meta-Analysis

**DOI:** 10.1186/s40798-018-0137-0

**Published:** 2018-06-07

**Authors:** Katherine J. Ombrellaro, Nita Perumal, Johannes Zeiher, Jens Hoebel, Till Ittermann, Ralf Ewert, Marcus Dörr, Thomas Keil, Gert B. M. Mensink, Jonas D. Finger

**Affiliations:** 1 Department of Epidemiology and Health Monitoring, Robert Koch Institute, Berlin, Germany; 2 Institute of Tropical Medicine and International Health, Charité - Universitätsmedizin Berlin, Berlin, Germany; 3 Institute for Social Medicine, Epidemiology and Health Economics, Charité - Universitätsmedizin Berlin, Berlin, Germany; 4 Institute for Community Medicine, University Medicine Greifswald, Greifswald, Germany; 5 Department of Internal Medicine B - Cardiology, Intensive Care, Pulmonary Medicine and Infectious Diseases, University Medicine Greifswald, Greifswald, Germany; 6 DZHK (German Centre for Cardiovascular Research), partner site, Greifswald, Germany

**Keywords:** Cardiorespiratory Fitness, Socioeconomic Status, Education, Adults, Social Health Inequality, Health Monitoring, Meta-Analysis, Systematic Review

## Abstract

**Background:**

This review aims to (1) consolidate evidence regarding the association between socioeconomic status (SES) and cardiorespiratory fitness (CRF), (2) conduct a meta-analysis of the association between SES and CRF using methodologically comparable data, stratified by sex, and (3) test whether the association varies after adjustment for physical activity (PA).

**Methods:**

A systematic review of studies from MEDLINE, EMBASE, Latin American and Caribbean Health Sciences (LILACS), Scientific Electronic Library Online (ScIELO), and Cochrane Library without time or language restrictions, which investigated associations between SES and CRF. Risk of bias within studies was assessed using a customized quality assessment tool. Results were summarized in table format and methodologically similar studies were synthesized using meta-analysis of Hedges’ *g* effect sizes. Synthesized results were appraised for cross-study bias. Results were tested for the impact of PA adjustment using meta-regression.

**Results:**

Compared to individuals with low education, both men and women showed higher CRF among individuals with high education (men 0.12 [0.04–0.20], women 0.19 [0.02–0.36]), while participants with medium education showed no significant difference in CRF (men 0.03 [− 0.04–0.11], women 0.09 [− 0.03–0.21]). Adjustment for PA did not significantly impact the association between education and CRF.

**Conclusions:**

There is fair evidence for an association between high levels of education and increased CRF. This could have implications for monitoring, of health target compliance and of chronic disease risk among higher risk populations, to detect and prevent non-communicable diseases (NCDs) and to diminish social health inequalities.

**Trial Registration:**

PROSPERO, CRD42017055456

## Key Points

- Systematically reviewed studies predominantly observed a positive association between socioeconomic status and cardiorespiratory fitness among men and women.
- The meta-analysis of the most frequently reported association between education and cardiorespiratory fitness showed a significant positive association for men and women when comparing the highest with the lowest of three education groups.
- Adjustment for physical activity did not affect the association between education level and cardiorespiratory fitness in the meta-analysis.

## Background

In 2005, chronic disease deaths were double the number of deaths resulting from infectious diseases (HIV/AIDS, TB, and malaria), maternal and perinatal conditions, and nutritional deficiencies combined [1]. Similarly, in 2015, 40 million or 70% of all-cause deaths globally were a result of chronic disease [2], a figure expected to increase to 52 million non-communicable disease (NCD) deaths by 2030 [3].

Socioeconomic status (SES), as defined by education, occupation, and income [4] plays a major role in the distribution of NCDs [5]. Evidence from high-income countries shows NCD burden effectively shifts to those with lower SES over time [6, 7]. Potential shift of disease burden to the poor, paired with increasing NCD and communicable disease burden on clinical and prevention resources means that individuals from lower SES groups may receive inadequate care, making them a priority for early prevention and monitoring. In fact, the World Health Organization (WHO) ranks monitoring and surveillance of risk factors as a top priority to tackle growing NCD epidemics in low-resource settings [8].

There is clear consensus in the literature that cardiorespiratory fitness (CRF), or “the ability of the circulatory and respiratory systems to supply oxygen during sustained physical activity (PA)” ([9], p. 53), measured at gold standard as maximal oxygen output, or VO_2__max_ obtained during maximal treadmill or ergometer testing [10, 11], is as important as PA [12–14], if not more important [15], for the prediction of future adverse health outcomes, including adverse cardiovascular events and all-cause mortality [16]. CRF is also often an objective measure of fitness, while PA, defined as bodily movement produced by skeletal muscles that require energy expenditure [9], is often self-reported behavior. The objective nature of CRF testing makes it the most reliable test of fitness for use in large-scale, population-based studies. Furthermore, directly measured fitness is more strongly associated with a protective cardiovascular risk profile than self-reported PA level [17], helping practitioners more accurately separate individuals with high long-term risk (25 years) for NCDs from those with low long-term risk.

As clinical and preventive resources stretch to meet increasing disease burden, it becomes essential to invest in interventions for early detection and treatment of NCDs, thereby reducing the need for additional or more expensive treatment in the future, and long-term economic burden [18]. Establishment of a relationship between SES and CRF may be helpful in accurately targeting the most at-risk groups for timely NCD prevention and early detection and treatment. To our knowledge, there are currently no systematic reviews addressing the relationship between SES and CRF in the general, adult population.

The overall aim of this systematic review is to (1) review and consolidate evidence from the literature regarding the association between SES and CRF, (2) conduct a meta-analysis of the association between SES and CRF using methodologically comparable data sources, stratified by sex, and (3) test whether association varies with adjustment for PA using meta-regression. We stratify by sex because sex differences in CRF are well documented [19–21] but also because identifying and addressing gender inequality in health is a priority for international health professionals [22]. We also test for the effect of adjustment for PA, because PA partially, but not exclusively [23], leads to CRF [24–27] and may influence the relationship between SES and CRF.

## Methods

### Protocol and Registration

This review was conducted as part of a larger research project investigating the personal and interpersonal correlates and/or determinants of CRF in adults. It is a subset of a broader systematic review that was registered at International Prospective Register of Systematic Reviews (PROSPERO): CRD42017055456. The systematic review protocol was published elsewhere in detail [28]. Instead of all determinants and correlates of CRF, the current review focuses on the association between SES and CRF.

### Literature Search and Selection Criteria

We conducted our search for journal-published articles in the MEDLINE (1965 to present), EMBASE (1947 to present), Latin American and Caribbean Health Sciences (LILACS, 1982 to present), Scientific Electronic Library Online (ScIELO, 1998 to present), and Cochrane Library literature databases. We additionally searched the Google Scholar grey literature database. In addition to electronic literature databases, the reference lists of all articles selected for full-text screening were hand searched for relevant studies not found in the electronic database search. The final database search was updated on October 30, 2017.

No date, language, article type, or text availability filters were applied. All search results were imported into the reference management software, Endnote X7 (Thomas Reuters, USA), and duplicates were removed. The current review includes quantitative observational (cohort studies, case-control studies, and cross-sectional studies) and experimental studies that report on the association between SES and CRF in the general adult population.

Eligible SES indicators were any acknowledged resource or prestige-based measure of position within a societal structure [3, 29] defined according to the MeSH (medical subject headings) term “Socioeconomic Factors” and equivalents. The Socioeconomic Factors MeSH term includes sub-headings such as educational status, employment status, income, occupation including career mobility, poverty including poverty areas (defined as city, urban, rural, or suburban areas which are characterized by severe economic deprivation and by accompanying physical and social decay), family characteristics (including family demography and family life surveys), social change, social class including social mobility and social conditions. Individual, household and area-based SES indicators as well as social mobility indicators were included.

Eligible CRF indicators were any acknowledged objective measures of CRF derived from maximal or submaximal incremental cardiopulmonary exercise testing (CPET) on a treadmill or cycle ergometer. Oxygen consumption indicators, either directly measured with spiroergometric gas exchange measurements or indirectly estimated with metabolic equations, were included. Maximal oxygen consumption (VO_2__max_) is defined as the oxygen consumption, in millimeter/(kilogram per second), during exercise, at which actual oxygen consumption reaches a maximum which cannot be increased with an increase in effort (plateau), while peak oxygen consumption (VO_2__peak_) is the highest VO_2_ value obtained on a particular test, regardless of the subject’s effort [30, 31]. Throughout the following, we will use the abbreviation VO_2__max,_ for both VO_2__max_ and VO_2__peak_ indicators. In addition to VO_2__max_, the following CRF indicators were included: physical working capacity in watts at variable and fixed heart rate thresholds (e.g., PWC_75%,_ PWC_170_), time in seconds to heart-rate threshold (e.g., WL_130_), energy expenditure in METs (metabolic equivalents), and total exercise duration in seconds.

The following exclusion criteria were applied: (1) studies measuring the impact of interventions designed to increase PA or CRF; (2) studies including only children, or adolescent participants (0–18 years) or elderly participants (90 years or older); (3) studies with sample sizes of less than 300 participants (considered too small to be representative of the general adult population [32], and the minimum sample size required for precise estimates of population mean differences [33]); (4) studies where participants were not representative of the general adult population (e.g., highly select populations, individuals from occupational groups with elevated PA, such as military groups or firefighters, symptomatic, or institutionalized individuals; (5) studies reporting only measures of childhood SES, such as family demographics and indicators found in family life surveys (because these SES measures are family based and do not always reflect an individual’s own SES in adulthood); and (6) reviews, letters to the editor, commentaries, or editorials.

Two reviewers (NP, KO) independently reviewed titles and abstracts of all references identified from databases and additional literature sources. Articles that were not excluded at this stage were further reviewed for inclusion, based on the publication’s full text, by reviewers (NP, KO); disagreements were resolved by a third reviewer (JF). Additional details about study selection are published elsewhere [28]. At all stages, disagreement between first and second reviewers was resolved by discussion. All studies examining the association between participant SES and CRF were included for data extraction and systematic review. In some cases, population-based studies had measured but not reported participant SES (*n* = 4). These studies were neither excluded, nor extracted, but were reserved for author follow-up. Articles based on population-based studies that were reserved for author follow-up were only included for systematic review if authors responded with supplementary data. All other studies were excluded from the systematic review. Studies included for meta-analysis were only those included for systematic review with directly comparable exposures of interest.

### Data Coding and Assessment of Methodological Quality

Studies were coded for study characteristics, methods, population characteristics, exposures and outcome variables, main results including method of analysis and confounders adjusted, as well as major limitations reported by the authors.

Supplementary details about data extraction process are published elsewhere [28]. All data were extracted by two reviewers (KO and JZ). In several cases, we contacted authors requesting additional data. Additional author requests were made when studies presented insufficient measure of the association between SES and CRF, or when population-based studies that were reserved for full-text screening, measured, but did not present data on the association SES and CRF.

Risk of bias within each study was independently assessed by two reviewers (JZ, KO) using a customized version of the Quality Assessment Tool for Observational Cohort and Cross-Sectional Studies by the National Heart, Lung and Blood Institute at the National Institutes of Health, USA [34]. Risk of bias was categorized as “high” when a study reached ≤ 49% “requirement fulfilled” score, “moderate” when a study reached 50–75% “fulfillment” score, and “low” at ≥ 75% “fulfillment” score. Supplementary details about risk assessment procedure are published elsewhere [28], results of the risk of bias assessment are available in Table 1. Additional sensitivity analysis, using risk of bias score to test the effect of study quality on the association between SES and CRF, was to be conducted if methodologically similar studies, included for meta-analysis, varied in risk of bias score.

**Table 1:** Customized risk of bias assessment—risk of bias was categorized as high when < 49% requirements met, moderate when 50–75% requirements met, and low when > 75% requirements met

	Question	Blair [46]	Braun [44]	Sidney [48]	Ceaser [47]	Finger [52]	Shmueli [50]	Fogelholm [43]	Lindgren [53]	Thai [49]	Dyrstad [42]	Cleland [51]	Lakka [41]	MacAuley [128]	Ittermann [45]	Shishebor [54]
1	Was the research question clearly stated?	1	1	1	1	1	1	1	1	1	1	1	1	1	1	1
1a	Were the correlates being investigated clearly stated?	1	1	1	1	1	1	1	1	1	1	1	1	1	1	1
1b	Was the CRF outcome clearly stated?	1	1	1	1	1	1	1	1	1	1	1	1	1	1	1
2	Was the study population clearly defined?	1	1	1	1	1	1	1	1	1	1	1	1	1	1	1
3	Study participant selection															
3a	Was a probability-based sampling strategy used?	0	1	1	1	1	0	0	0	1	0	1	0	1	1	0
3b	Was sampling frame at a national level?	0	1	1	1	1	0	1	0	1	1	1	0	1	1	0
3c	If participants were selected from clusters, were there ≥50 clusters?	0	1	1	1	1	0	0	0	1	0	1	NA	NR	0	0
3d	Were inclusion and exclusion criteria pre-specified?	1	1	1	1	1	1	1	1	1	1	1	1	1	1	1
4	Was the sample size greater than 1200?	1	1	1	1	1	1	0	0	1	0	0	1	0	1	1
4a	Was sample size justification or power description provided?	0	1	1	1	1	0	0	0	1	0	0	0	NR	NR	0
5	Response rate															
5a	Was response rate of eligible participants > 50%?	NA	1	1	1	1	NA	1	0	1	1	1	1	1	1	NA
6	Loss to follow-up in cohort studies															
6a	Was loss to follow-up after baseline ≤20%?	NA	NA	NA	NA	NA	NA	NA	NA	NA	NA	0	NA	NA	NA	NA
7	Were correlates of CRF and potential confounders objectively measured using validated instruments?	1	1	1	1	1	1	1	1	1	1	1	1	1	1	1
7a	Were few exposure variables self-reported?	1	1	1	1	1	1	1	1	1	1	1	1	1	1	1
7b	Was the outcome variable clearly defined, valid, reliable, and reliable?	1	1	1	1	1	1	1	1	1	1	1	1	1	1	1
8	Was CRF measured using an objective, reliable, and validated methodology?	1	1	1	1	1	1	1	1	1	1	1	1	1	1	1
8a	Was CRF measured consistently for all participants?	1	1	1	1	1	1	1	1	1	1	1	0	1	1	1
9	Were the exposure variables measured prior to measurement of the outcome variables?	1	NR	NR	NR	1	NR	1	NR	1	NR	0	NR	NR	NR	1
10	Were key potential confounding variables measured and adjusted for statistically?	1	1	1	1	1	1	1	1	1	1	1	1	1	1	1
11	Did the study investigate interaction between exposure variables?	0	1	0	0	0	1	1	1	1	0	0	0	0	0	0
12	Did analysis include sensitivity analysis?	1	1	1	1	1	1	1	1	1	0	1	1	0	1	1
13	Was funding source and/or conflicts of interest reported?	0	0	1	1	1	0	1	1	1	1	1	1	1	1	0
	Final Assessment	63%	92%	92%	92%	96%	63%	73%	57%	100%	61%	77%	66%	71%	84%	63%
	Risk of Bias Evaluation	Medium	Low	Low	Low	Low	Medium	Medium	Medium	Low	Medium	Low	Medium	Medium	Low	Medium

Quality Assessment Tool for Observational Cohort and Cross-Sectional Studies by the National Heart, Lung and Blood Institute at the National Institutes of Health, USA

### Statistical Analysis

After completion of the author requests for additional data, results of the systematic review were summarized in table and narrative format. Comparable data was only obtained for the relationship between education and CRF (*n* = 3). In order to pool results for meta-analysis, we standardized education categories across population-based studies into three main categories according to the CASMIN educational classification—high, medium, and low [35]. We also standardized the outcome measurement using VO_2__max_, in millimeter/(kilogram per second), calculated according to the American College of Sports Medicine equation: 3.5ml × min^−1^ × kg^−1^ + 12.24 × *w*_max_ × bodyweight^−1^ [36, 37] or directly measured with Spiroergometry (ml/min). Individual study results were standardized using the Hedges’ *g* effect size [38] calculated as ES = (((mean difference between reference and comparison categories)/(pooled and weighted standard deviation)) × correction factor (*J*)) to determine the overall association between education and CRF. The standardized effect sizes were then included in a random effects meta-analysis according to the DerSimonian and Laird [39] methodology; this was pre-specified due to the expected heterogeneity of outcome and exposure measurements in the underlying studies and also because it was expected that the effect of SES on CRF varies by context, and therefore that analysis would estimate the distribution of these effects, rather than estimating one true effect of SES on CRF. Our final meta-analysis model adjusted for the set of confounders adjusted for in all studies: age, PA, waist circumference (WC), body mass index (BMI), and alcohol consumption. Data analysis was performed using STATA Version 14 statistical software (Stata Corporation, College Station, TX, USA). Increases in CRF were reported as positive values and high and medium educational categories were compared to the referent low educational category so that positive CRF illustrated improvement in the comparison over the referent education category. Data are reported as mean ± 95% confidence interval (CI).

The *I*^2^ statistic (percentage of variance in the study-specific point estimates that is attributable to true between-study heterogeneity as opposed to sampling variation) was used as an indicator of study heterogeneity or risk of bias across studies. Evidence of heterogeneity was determined by a *p* value < 0.1 [40], to address the low power of the statistical test resulting from limited number of studies included for meta-analysis. The meta-analysis was stratified by sex and included additional sensitivity analysis to test differences, in the synthesized association between SES on CRF, with and without adjustment for PA. Differences were tested using meta-regression. Previously mentioned additional sensitivity analyses were pre-specified. Post-hoc analysis adjusting NHANES data for race was performed.

## Results

### Study Characteristics

**Fig. 1:**
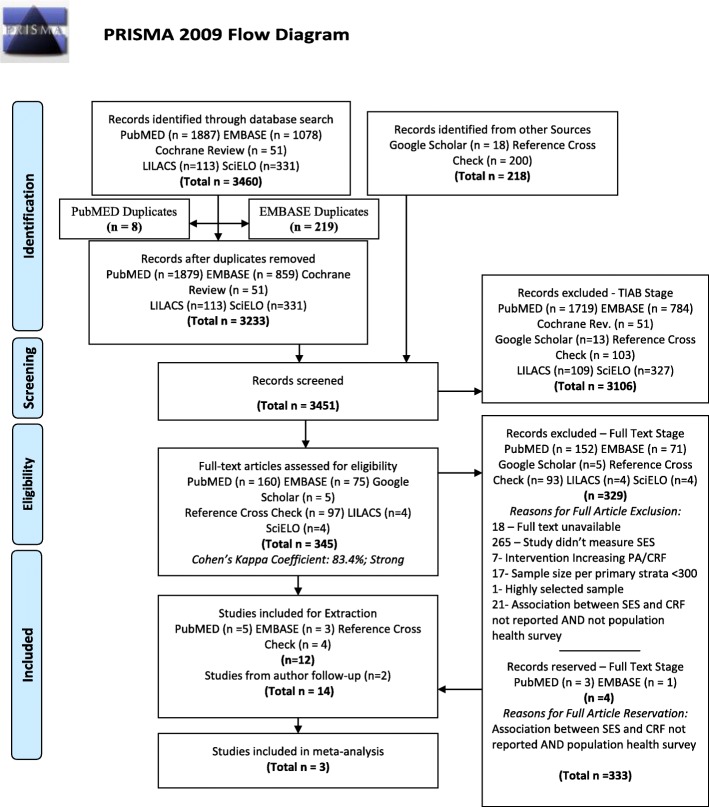
Search strategy: PRISMA flow diagram. TIAB, title abstract

A PRISMA flowchart depicting the article selection process can be found in Fig. 1. An updated search was conducted in October 2017, resulting in no new results. A total of 3233 studies were identified from electronic databases, and 218 articles were identified from additional literature sources. After title and abstract screening, 346 articles were included for full-text screening, of which 329 were subsequently excluded. Four articles reporting data from population-based studies were reserved for author follow-up because they measured, but did not report on the association between SES and CRF. In total, 15 studies were included for systematic review and three were included for meta-analysis, resulting in four population-based studies for meta-analysis (since one study contained two independent cohorts).

In our search we did not exclude experimental studies, although it is difficult to imagine examples of experimental studies designed to modify SES, in order to improve CRF. Ultimately, however, all included studies were observational, since no experimental studies fulfilled the eligibility criteria. The associations between SES exposures and CRF found in the systematic review (positive, negative, U-shaped (all variants), and not significant), are presented in Tables 2 and 3 according to socioeconomic exposure.

**Table 2:** Results summary: education

Year	Author	Design	Country	*n*	Age	CRF	Measure	Association	Direction	*p* value	Quality
1996	Lakka et al. [41]	CRF Cross-sectional	FIN (KIHD)	Male 2280	42–61	VO_2__max_ (l/min) cycle Protocol: Individual	Mean + SD	Positive gradient	+	*p* < .001	66%
2014	Ittermann et al. [45]	Cross-sectional	DE (SHIP-1) & (SHIP-Trend)	male 2074 female 2110	20–85	VO_2__max_ (ml/min) cycle Protocol: Standardized (modified Jones)	*β*	Higher in high vs. low education (m) CRF increases with education (f)	+	*p* < .009 (m) *p* < .056 (f)	84%
2005	Dyrstad et al. [42]	CRF cross-sectional	N	Male 900	18–19	VO_2__max_ (mL/kg∙min) cycle Protocol: Individual	Mean + SD	8% higher fitness in high school academic vs. vocational training programs	+	*p* < .01	61%
2013	Ceaser et al. [47]	Cross-sectional	US (NHANES)	Total 3245	20–49	VO_2__max_ (mL/kg∙min) treadmill Protocol: Standardized (NHANES)	*β*	Increases with education (Hispanic Americans)	+	*p* = .01	92%
2006	Fogelholm et al. [43]	Cross-sectional	FIN	Male 891	21–43	VO_2__max_ (mL/kg∙min) cycle Protocol:Individual	*β* ^a^	Increases with education	+	.01 < *p* < .05	73%
2014	Thai et al. [49]	Cross-sectional	US (NHANES)	Total 2761	12–49	Low eVO_2__max_ (≤ 31.98 mL/kg∙min) treadmill Protocol: Standardized (NHANES)	OR	Higher odds in medium vs. low education	U	OR 95% CI (1.01–1.97)	100%
2014	Shmueli et al. [50]	Cross-sectional	IL (TAMCIS)	Total 3854	20–80	CRF in METS treadmill Protocol: Standardized (Bruce)	*β*	Higher mean difference in medium. vs. low education	U	*p* < .05 *p* trend = .002	63%
1995	Braun et al. [44]	Cross-sectional	US (CARDIA)	Total 4930	18–30	Exercise duration (sec) treadmill Protocol: Standardized (modified Balke)	*β*	Increases with education	+	*p* < .05	92%
						WL_130_ (sec) treadmill Protocol: Standardized (modified Balke)	*β*	No association	Ø	NR	
1992	Sidney et al. [48]	Cross-sectional	US (CARDIA)	Black male 1123 white male 1147 black female 1428 white female 1270	18–30	Exercise duration (sec) treadmill Protocol: Standardized (modified Balke)	*β*	Increases with education (black male)		*p* < .05 (black male)	92%
								Higher (white male) highest (white female)	+	*p* < .001 (white male and female)	
								No association (black female)	NS	NS (black female)	
						WL_130_ (sec) treadmill Protocol: Standardized (modified Balke)	β	WL_130_ increases with education (black male)	+	*p* < .05 (black male)	
1984	Blair et al. [46]	Cross-sectional	US (Cooper Center)	Female 2200	18–75	Exercise Duration (sec) treadmill Protocol: Standardized (modified Balke)	*β* ^a^	Highest with postgraduate study, some college decreases duration most	U	*p* < .01	63%
Social mobility											
2009	Cleland et al. [51]	Prospective cohort	AU (CDAH)	Total 645	26–36	Fitness Decrease (PWC_170_) cycle Protocol: Standardized (W170)	RR	Higher risk of decrease in fitness than persistent unfit state in persistent medium vs. persistently low SES	(−)	*p* < .05	77%
						Fitness Persists (PWC_170_) cycle Protocol: Standardized (W170)	RR	Higher risk that unfit state persists than fitness persists in persistent medium vs. persistently low SES	(−)	*p* < .05	
						Fitness Increase (PWC_170_) cycle Protocol: Standardized (W170)	RR	Higher likelihood that fitness increases than unfit state persists in high and upwardly mobile vs. persistently low SES	+	*p* < .05	

^a^Standardized beta coefficient

**Table 3:** Results summary: additional socioeconomic status indicators

Year	Name	Design	Country	*n*	Age	CRF	Measure	Association	Direction	*p* value	Quality
Socioeconomic indicator: composite SES index											
2014	Shmueli et al. [50]	Cross-sectional	IL (TAMCIS)	Total 3854	20–80	CRF in METS treadmill Protocol: Standardized (Bruce)	*β*	CRF increases with SES	+	*p* < .05 *p* trend < .001	63%
1998	MacAuley et al. [128]	Cross-sectional	IE (NIHAS)	Total 528	16–74	VO_2__max_ (mL/kg∙min) treadmill Protocol: Individual	Mean + SD	No association	Ø	NS	71%
2013	Finger et al. [52]	Cross-sectional	DE (DEGS)	Male 1371 female 1441	18–64	High CRF (top 40% of PWC_75%_ dist.) cycle Protocol: Standardized (WHO)	OR	CRF increases with SES (f)	+	*p* < .001	96%
Socioeconomic indicator: residential area SES											
2016	Lindgren et al. [53]	Cross-sectional	S (SCAPIS)	Total 592	50–64	VO_2__max_ (mL/kg∙min) cycle Protocol: Standardized (SCAPIS)	median + (IQR)	Median fitness increases with area SES	+	*p* < .05	57%
2008	Shishehbor et al. [54]	Cross-sectional	US (CARDIA)	Total 2505	25–42	Impaired CRF in METs (lowest quintile per gender) treadmill Protocol: Standardized (modified Balke)	OR	Lower odds of low fitness in high SES areas	+	*p* trend < .001	63%
Socioeconomic indicator: occupation											
1996	Lakka et al. [41]	CRF cross-sectional	FIN (KIHD)	Male 1907	42–60	VO_2__max_ (l/min) cycle Protocol: Individual	mean + SD	Higher mean fitness among white collar than blue collar workers and farmers	+	*p* < .001	66%
2014	Shmueli et al. [50]	Cross-sectional	IL (TAMCIS)	Total 3854	20–80	CRF in METS treadmill Protocol: Standardized (Bruce)	β	Fitness mean difference increases with occupation skill	+	*p* < .05 *p* trend < .001	63%
Socioeconomic indicator: income											
1996	Lakka et al. [41]	CRF cross-sectional	FIN (KIHD)	Male 1907	42–60	VO_2__max_(l/min) cycle Protocol: Individual	Mean + SD	Positive gradient	+	*p* < .001	66%
2014	Shmueli et al. [50]	Cross-sectional	IL (TAMCIS)	Total 3854	20–80	CRF in METS treadmill Protocol: Standardized (Bruce)	*β*	No Association	Ø	NS	63%
Socioeconomic indicator: employment status											
1996	Lakka et al. [41]	CRF cross-sectional	FIN (KIHD)	Male 2280	42–60	VO_2__max_ (l/min) cycle Protocol: Individual	Mean + SD	Higher mean fitness when employed	+	*p* < .001	66%

Studies were cross-sectional (*n* = 14) and cohort (*n* = 1). Sample sizes ranged from 528 to 4968 participants. Studies included participants aged 16–85 years. Studies were conducted across a span of 41 years—from 1971 to 2012; four were conducted between 1971 and 1990; one study spanned from 1985 to 2006; eight were conducted between 1992 and 2011; and two contained end points after 2011. Studies were from the US (*n* = 6), Finland (*n* = 2), Germany (*n* = 2), Norway (*n* = 1), Sweden (*n* = 1), Ireland (*n* = 1), Israel (*n* = 1), and Australia (*n* = 1). Most common confounders adjusted for were age, PA, alcohol consumption, BMI, and WC. One study fulfilled all methodological quality criteria, six studies had low risk of bias, and eight studies had moderate risk of bias. The major risk of bias across studies was participant selection methodology i.e. sampling method other than probability-based sampling.

#### Outcome: CRF

Included studies (*n* = 15) were heterogeneous with respect to measurement of CRF. CRF was measured and reported as estimated VO_2__max_ (ml/kg min) in six studies, while two studies directly measured VO_2__max_ using breath analysis (l/min). Three studies measured and reported CRF as exercise duration (seconds), in some cases additionally paired with a heart-rate indicator (WL_130_). Two studies measured and reported METS (energy expenditure during treadmill testing). Indicators reported by only one study include PWC_75%_ (physical working capacity at 75% of the predicted maximal heart rate, watts) and longitudinal fitness categories constructed using PWC_170_ (watts). Specific details about CRF measurement can be found in Tables 2 and 3.

#### Exposure: SES Indicators

Categorical education (years), the most frequent indicator, was presented in 11 studies; 10 studies reported own education, and 1 study reported longitudinal educational mobility categories. Other SES exposures included composite measures of SES combining several indicators (high, medium, low, *n* = 3), residential area-level SES (high, medium, low, *n* = 2), own occupation (*n* = 2), income based indicators (*n* = 2), and employment status (employed or unemployed, *n* = 1).

### Results of Individual Studies by Exposure

Socioeconomic exposures excluded from meta-analysis generally showed a positive relationship between SES exposure and CRF measure of interest. Individual studies within the primary exposure for meta-analysis, education, generally showed a positive relationship between high education and CRF measure of interest. Three studies showed a u-shaped relationship.

#### Education

Four studies [41–44] observed a positive association between education and VO_2__max_ (*p* < 0.05, 0.01 < *p* < 0.05, *p* < 0.01, *p* < 0.001). The study [45] observed that VO_2__max_ increased with education among women (*p* < 0.056), but only high education improved CRF relative to low education among men (*p* < 0.009). Exercise duration from one study [46] increased most when comparing the highest and lowest education categories (*p* < 0.01). Two studies [47, 48] presented a positive association between education and CRF that varied by ethnic subgroup. Study [47] observed a significant positive association between education and CRF, among Hispanic Americans only (*p* = 0.01). The study [48] observed higher positive association between education and exercise duration among white participants *(p* < 0.001), compared to black participants (*p* < 0.05). The increase in exercise duration with education was higher among white men and highest among white women but was non-significant among both subgroups for WL_130_. Black males showed increase in WL_130_ with education (*p* < 0.05), while black women showed no significant associations for either measure of CRF.

Three studies [46, 49, 50] observed a u-shaped association between education and CRF. Among the studies reporting an inverted u-shaped association, study [50] observed that CRF increase was largest when comparing medium and low education level (*p* < .05). Study [49] presented an OR measure of association between education and CRF and observed that participants in medium education group had higher odds of *low* VO_2__max_ (OR 1.41, 95% CI (1.01–1.97)), than participants in the high education group (OR 1.24, 95% CI (0.79–1.94)), when compared to lowest education group. This study was additionally adjusted by measures of periodontal health.

The study [51] observed an association between social mobility and longitudinal fitness. The study observed that persistently high or upwardly mobile SES status, compared to the persistently low SES status, resulted in higher likelihood of increased fitness (*p* < 0.05) than persistence of an unfit state.

#### All other SES Indicators

Studies [50, 52] reporting a significant association between CRF and composite socioeconomic indices presented multivariable analysis and observed a positive association (*p* < 0.05, *p* < 0.001). Results from the study [52] varied by sex; odds of high fitness were increasingly greater (*p* < 0.001) with higher SES index score, among women, while men showed non-significant results.

Studies [53, 54] reporting on the association between CRF and residential area SES conducted multivariable regression analysis and observed that median VO_2__max_ increased and odds of low fitness (METs) decreased with higher residential area SES.

Both studies [41, 50] reporting the association between participant occupation and CRF observed a significant positive association between skilled occupation and CRF (*p* < 0.001 and *p* < 0.05).

While study [50] observed no significant association between financial strain and METS during treadmill exercise, study [41] identified a positive linear association between income and VO_2__max_ (*p* < 0.001) using ANCOVA analysis.

Finally, the study [41] observed that VO_2__max_ was higher among employed individuals (*p* < 0.001).

### Direct vs. Indirect VO_2__max_ Measurement

Across all exposures, studies measuring and reporting direct measures of VO_2__max_ showed a strictly positive relationship between SES and CRF, while indirect measures of VO_2__max_ showed a positive relationship overall. Two studies directly measured VO_2__max_ through breath analysis and reported a positive association between SES and CRF. Among studies estimating VO_2__max_, four studies reported a positive association, one reported an inverted u-shaped association and one reported no association.

### Synthesis of Results

**Fig. 2:**
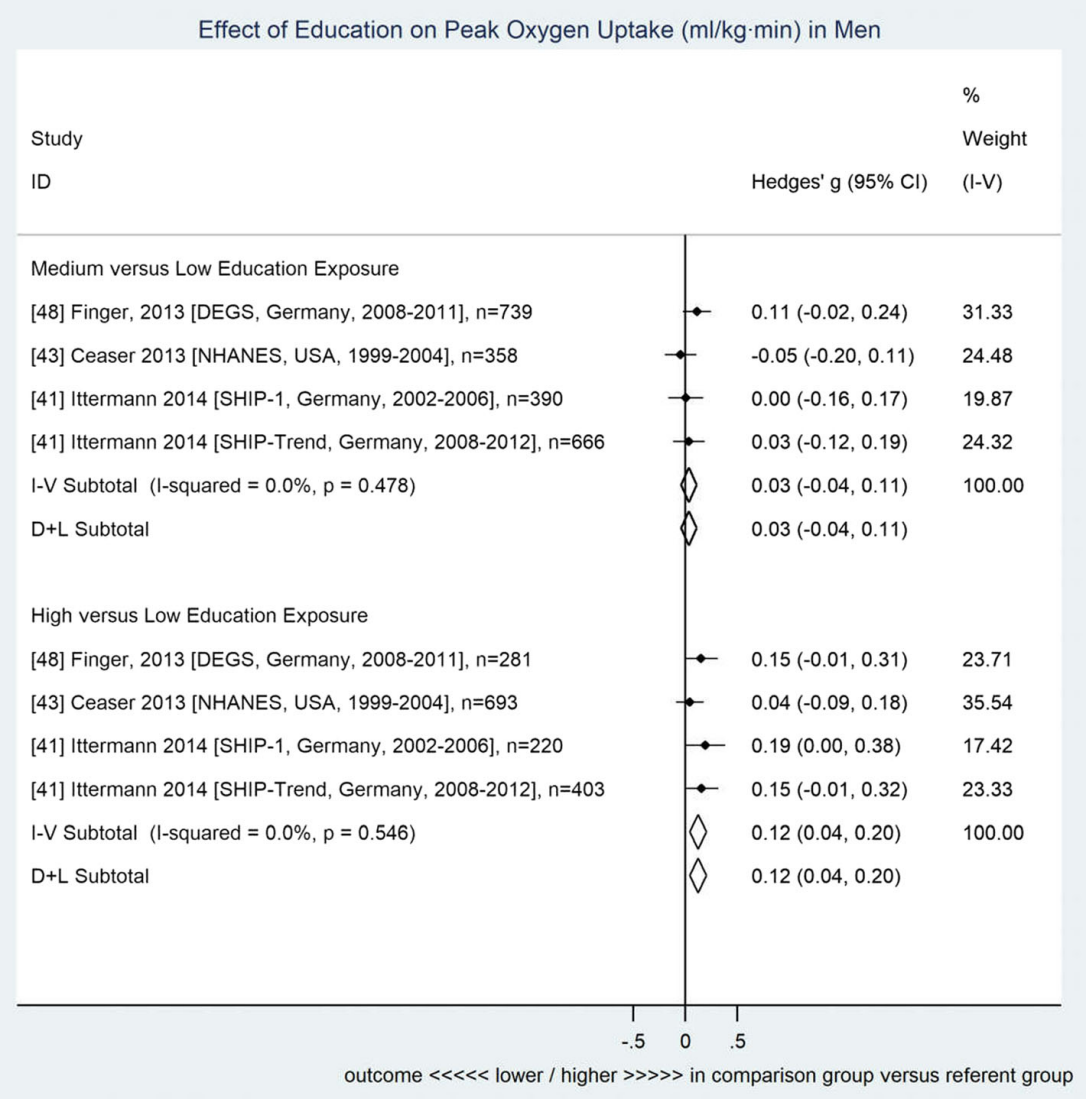
Forest plot for the association between education and cardiorespiratory fitness among men. Data shown are standardized mean differences ± 95% CI (fully adjusted including physical activity, *n* = 4815). Subtotals presented for both fixed (inverse variance method) and random effects (DerSimonian and Laird) models. Reference details precede study descriptors

**Fig. 3:**
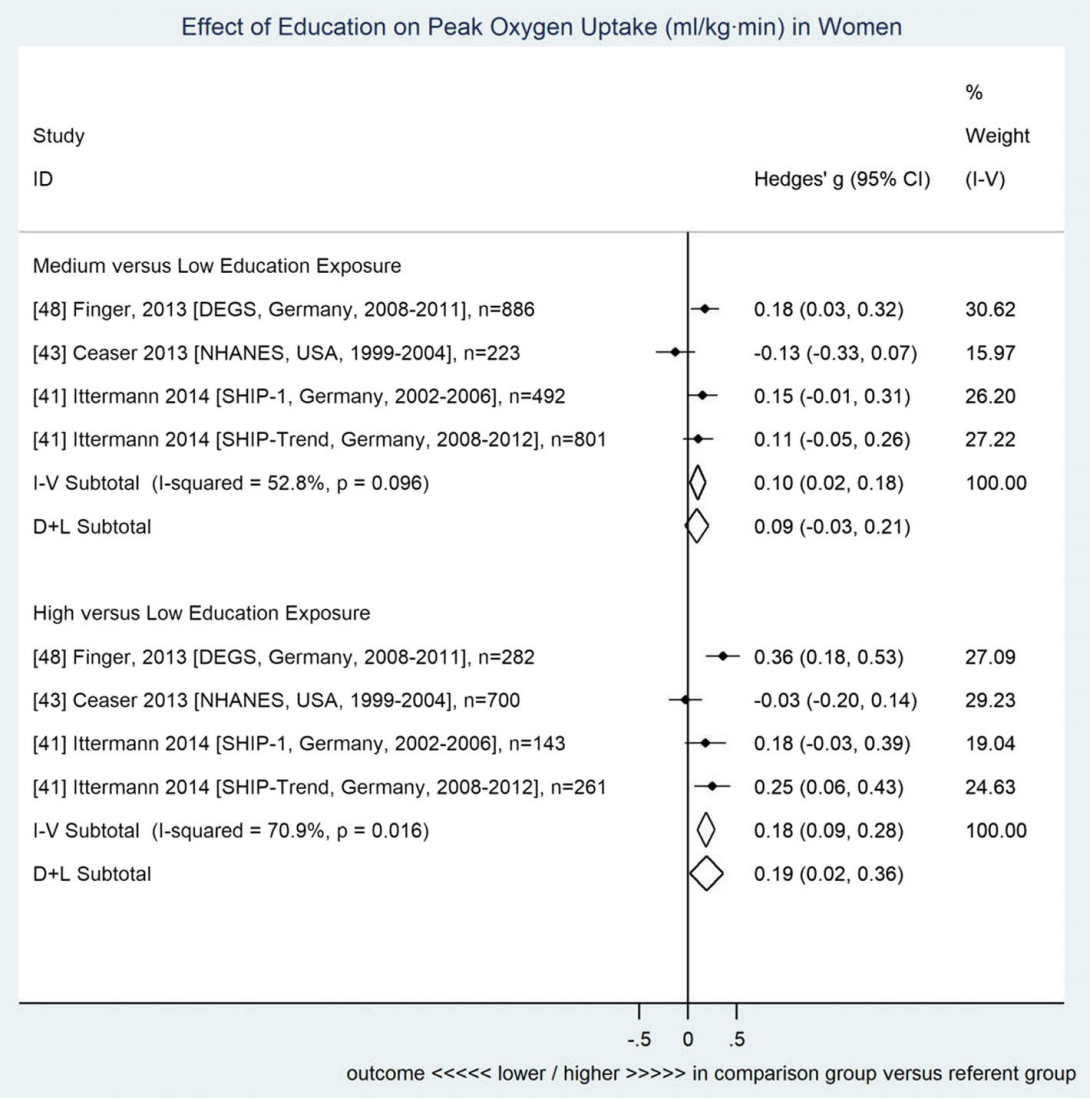
Forest plot for the association between education and cardiorespiratory fitness among women. Data shown are standardized mean differences ± 95% CI (fully adjusted including physical activity, *n* = 4620). Subtotals presented for both fixed (inverse variance method) and random effects (DerSimonian and Laird) models. Reference details precede study descriptors

Results of meta-analysis are presented in Figs. 2 and 3. Compared to individuals with low education, both men and women with high education showed significantly higher CRF (men 0.12 [0.04–0.20], women 0.19 [0.02–0.36]), while participants with medium education showed no significant difference in CRF compared to individuals with low education (men 0.03 [− 0.04–0.11], women 0.09 [− 0.03–0.21]).

### Risk of Bias Across Studies: (*I*^2^ Measure of Heterogeneity)

Our analysis standardizes both exposure and outcome to limit heterogeneity. Accordingly, among men the fully adjusted model (adjusted for age, PA, alcohol consumption, WC, and BMI) had low heterogeneity, with a non-significant *p* value > 0.1 (medium education: *I*^2^ = 0%, *p* value = 0.477; high education: *I*^2^ = 0%, *p* value = 0.544) while among women the fully adjusted model showed substantial heterogeneity, *p* value < 0.1 and *I*^2^ value in the range 50–90% [55] (medium education: *I*^2^ = 52.9%, *p* value = 0.095; high education: *I*^2^ = 71%, *p* value = 0.016). Presentation of the results from random effects meta-analysis adjusts for this heterogeneity within the fixed effects meta-analysis.

### Additional Analysis

Meta-regression testing differences in the effect of education on CRF with adjustment for PA detected no significant differences (*p* > 0.385).

Studies from the US, that were systematically reviewed, reported differences in the association between CRF and education by ethnicity of the study sample [48], thus we performed additional post-hoc sensitivity analyses, adjusting NHANES data by “race.” The measures of association between SES and CRF marginally increased, however the trend among men and women did not vary from the original meta-analysis. Studies included for meta-analysis had low risk-of-bias-score; thus, no sensitivity analysis by quality assessment score was conducted.

## Discussion

In this systematic review and meta-analysis of the association between SES and CRF in adults, evidence from 15 population-based studies from 8 different countries, shows that predominately higher SES is associated with increased CRF. Socioeconomic exposures, such as SES indices, composed of various SES indicators [50, 52], and residential area SES [54], generally showed a positive relationship with CRF [41, 53]. Studies using education level as an exposure, showed either a positive relationship between education and CRF [41–45, 47, 48, 51] or a u-shaped relationship [46, 49, 50].

Meta-analysis of the most frequently reported association; between education and VO_2__max,_ was based on a sample of 9435 non-symptomatic individuals from four population-based studies. Meta-analysis showed a significant positive association between education and CRF for men and women when comparing the highest with the lowest of three education groups. To the best of our knowledge, this is the first systematic review conducted on the association between SES and CRF; thus, it is impossible to compare our findings with previous reviews. However, reviews on the association between SES and PA report observations in line with our findings: a positive association between SES and health-enhancing total leisure time PA [56]. Additionally, research from both the USA and Germany shows that SES is positively associated with aerobic physical activity. In 2014, the percentage of US adults, age 18 and over, who met federal guidelines for aerobic physical activity increased as family income increased [57], with 51.7% of US adults meeting the 2008 federal physical activity guidelines for aerobic activity [58]. Similar patterns can be observed among German adults in 2014; 45.3% of German adults met the WHO recommendation for aerobic activity, with higher compliance among individuals with higher education [59].

CRF inequalities across levels of SES likely stem from differences in health behavior. Lower SES is associated with health-compromising behaviors such as low levels of aerobic leisure-time PA [56, 59, 60], high sugar-rich and fat-rich food intake and low fruit and vegetable intake [52, 61, 62], and high smoking prevalence [63–66]. While the previously mentioned health-compromising behaviors are strictly negatively associated with SES, the association between SES and alcohol consumption varies by dose. Heavy episodic alcohol consumption, defined as pure alcohol intake of 60 g or more, during a single occasion, at least once per month [67], is associated with lower SES [68], while risky alcohol consumption, or consumption of 10–12 g of pure alcohol daily for women and 20–24 g for men [69, 70] is associated with higher SES [71]. Lower SES is also related to obesity [72]. These disadvantageous behaviors and conditions lead to poorer health and are primary risk factors for chronic diseases such as diabetes [73–76], cardiovascular disease (CVD) [71, 76–80], and cancer [76–78, 81, 82]. Similarly, it has been demonstrated that obesity and overweight [56], physical inactivity, and smoking are negatively associated with CRF [83]. Conversely, moderate average alcohol consumption (defined as 4–15.8 g/d) improves CRF more than non- or heavy average alcohol consumption, in an inverted u-shaped fashion [84]. Overall, it is likely that these health behaviors and conditions are underlying causes of the positive association between SES and CRF. It is also possible that the positive association between SES and CRF is explained by the negative association between high SES and chronic breathlessness: individuals with high SES are less likely to suffer from chronic breathlessness and by extension to have higher CRF [85, 86]. Consider that 15% of participants from SHIP-0 (1997–2001; *n* = 4308) and 17.7% of participants from SHIP-Trend (2008–2012; *n* = 4420) reported “shortness of breath at load” [81], demonstrating that measured fitness may have been impacted by chronic breathlessness. Apart from behavioral and health-related factors, genetic factors are also known to influence physical fitness [87–91]. However, whether the association between SES and CRF could be partly explained by genetic dispositions cannot be determined based on available evidence in the literature.

The importance of CRF for public health is reflected in the policy statement from the American Heart Association, from 2013, calling for a national registry on CRF [92]. Previous research has demonstrated that increased CRF is associated with various health benefits leading to a significant reduction in mortality rates [93]. CRF can be increased through regular PA participation [94, 95], however, not all types of PA are beneficial for CRF. Occupational PA often corresponds with muscle-strengthening activity or low-intensity tasks performed over long periods (8-h work shifts) [96] and seems to be less beneficial for CRF than aerobic sports and physical exercise activities mostly performed during leisure time [41, 97]. Adults with low SES are more likely to work in physically-demanding jobs and to show a higher total energy expenditure compared to adults with high SES who are more likely to have sedentary jobs and perform aerobic physical exercise in leisure time [59, 60, 98]. Thus, it seems that adults with low SES do not show lower CRF because they are less physically active [99], rather, because the types of PA they perform are less often aerobic and hence less beneficial for CRF and cardiovascular health [100, 101]. As a result, consideration of SES differences in working conditions is essential to address SES differences in CRF. Health interventions, striving to improve PA at the population level, mostly promote aerobic PA in leisure time, and thus fail to reach adults with low SES. The low prevalence of aerobic PA in leisure time among individuals with low SES is also illustrated by increasing social inequality in sporting activity prevalence in the adult German population over the last decade [102]. Health promotion activity delivery to individuals with low SES backgrounds remains a crucial challenge, however, workplace aerobic physical activity interventions for manual workers are a possible solution to the challenge of reaching individuals, of low SES background, for CRF improvement [103]. In 2008, the US Federal Government issued *Physical Activity Guidelines for Americans* [104], which provided science-based guidelines recommending adult aerobic PA targets for achievement of substantial health benefits [105, 106], which were adapted by the WHO in 2010. The population-based monitoring of PA guideline compliance is difficult because PA is often monitored based on self-reports, making it difficult to distinguish aerobic PA from other types of PA, and introducing the possibility of misclassification bias. Objectively measured CRF, applied for population-based health monitoring purposes, can be an important tool to accurately gauge health target compliance and prevent bias from self-reported PA. Furthermore, objectively measured CRF can be used to monitor chronic disease risk, including cardiorespiratory disease risk [107]. CRF is an important tool for population health monitoring precisely because there is a large body of evidence that CRF is a potentially stronger predictor of mortality than established risk factors such as smoking, hypertension, high cholesterol and diabetes type 2 mellitus [108]. Furthermore, the addition of CRF to traditional risk factors significantly improves the precision of risk prediction for cardiovascular morbidity and mortality [109–111] and addition of CRF to traditional CVD risk measures (such as Framingham risk score or SCORE Risk Charts) improves cardiovascular risk prediction [112]. Clinicians use measures linking CRF changes to disease decline [16] to objectively monitor individual and population health risk. Clinicians could also use CRF thresholds [113] by education status to identify low SES groups suffering health disparity for targeted, early NCD prevention, potentially reducing the need for complex, expensive treatments and long-term economic burden. Insights about the association between SES and CRF could be used to monitor, prioritize and, by extension, improve health outcomes among marginalized populations with high risk of chronic disease, whose needs may not be met by traditional health promotion activities. Monitoring and prioritization of health outcomes among marginalized populations has been established by organizations such as WHO and the Pan American Health Organization (PAHO) as a key priority for controlling NCD epidemics in low resource settings.

### Limitations

Although included studies are generally population-based and not underpowered, the current meta-analysis includes only four population-based studies, due to the limited number of population-based studies reporting objective measures of CRF in the literature. Accordingly, the power of meta-analysis to detect a significant effect between SES and CRF may be limited. Ability to detect differences in effect by sex, with PA adjustment, and by ethnicity in NHANES data may also be limited by sample size of the meta-analysis. Our choice of education categories may have also affected results. The chosen education categories (high, medium, and low, based on CASMIN education classification [35]), may have limited the ability to detect subgroup differences through sensitivity analysis due to combination of disparate subgroups. Furthermore, overall results among women should be cautiously interpreted due to the high heterogeneity within this subgroup. Differences in the results of studies included for meta-analysis, and the resulting heterogeneity may be due to use of different exercise protocols for CRF measurement [114]. The association between CRF and various socioeconomic exposures was presented in the literature, but the present meta-analysis focuses on education due to issues with heterogeneity of exposure indicators used and minimum sample size required for rigorous meta-analysis. However, the omission of additional SES measures in the meta-analysis does not significantly impact overarching findings because SES indicators measuring different aspects of social position show similar association with CRF. For example, Shmueli et al. observed significantly different mean exercise capacity in higher vs. lower SES levels across education, occupation, and compiled SES indicators [50]. Similarly, Lakka et al. observed a positive dose relationship between education and income, and higher VO_2__max_ with higher occupational skill [41]. Although few studies report degree of agreement between association of various SES indicators, measuring different aspects of social position, with CRF, overall agreement between indicators can also be seen for the relationship with PA [56, 115]. Studies included for review adjusted their analysis for varying sets of covariates which may impact overall result agreement. We correct for this through meta-analysis of standardized effect sizes that were derived from individual study results, which had been adjusted for a standard set of covariates. Finally, generalizability across levels of country income classification may be limited due to inclusion of only studies from high-income countries. However, inclusion of studies from only high-income countries also reduces heterogeneity within the meta-analysis by controlling the effect of country income classification on the association between SES and CRF [116, 117].

### Recommendations

Systematic review of the literature revealed that few population-based studies reported SES exposures in addition to education. Population-level investigation of the effects of additional measures of SES, such as income, occupation, or composite SES indices on CRF is also necessary. Future research should include additional SES indicators in meta-analysis in order to gauge whether the relationship observed between education and CRF is generalizable to other SES indicators. Investigation of differences in the relationship between SES and CRF by outcome measure is also necessary, to compare the effect of SES on VO_2__max_ (gold standard) with the effect of SES on additional CRF measures commonly cited in the literature. Adjustment for total PA did not significantly impact the results of meta-analysis, however total PA obfuscates domain specific PA. Future research should investigate the effect of adjustment for domain specific PA types that are known to be differentially correlated with SES—such as occupational physical activity (correlated with low SES) and leisure time PA (correlated with high SES) [60]. Additionally, sedentary behavior is an important determinant of CRF [118, 119], but was not included as a covariate in analyses where CRF was the outcome of interest. Future research on the association between SES and CRF might include sedentary behavior as a study covariate to strengthen results. While the patterns observed for the association between education and CRF were fairly similar among men, differences in the association between education and CRF among women from Germany and the USA should be explored. Furthermore, although sensitivity analysis showed no significant difference in the effect of SES on CRF by ethnicity, additional research regarding the effect of ethnicity on the relationship between SES and CRF would contribute to more accurate monitoring of chronic disease risk within marginalized populations [120] and help to effectively target these groups for prevention [91, 121–123]. Most studies that were systematically reviewed were cross-sectional, thus more cohort studies are required to rigorously establish an association between SES and CRF. The meta-analysis disproportionately represents populations from Germany due to data access constraints, thus inclusion of population-based studies from various countries across high-income countries would improve result quality and external validity. Included studies are from high-income countries only; future research should consider whether low- and middle-income countries reflect the association observed in high-income countries, and whether nutritional and PA transition processes [124–127] that take place during economic development influence the association between SES and CRF.

## Conclusions

Despite limitations, we conclude that there is fair evidence in the literature for an association between high levels of education and increased CRF. This could have implications for monitoring, of health target compliance and of chronic disease risk among higher risk populations, to detect and prevent NCDs. In light of shifting NCD burden from adults with high SES to adults with low SES, defining CRF health targets, monitoring CRF and PA target compliance at the population level and developing tailored health promotion measures to stimulate CRF—especially among adults with low SES background—is necessary to improve cardio-metabolic health in the general adult population and to diminish social health inequalities.

Additional cohort studies are required to rigorously establish an association between SES and CRF. Furthermore, studies investigating the impact of ethnicity on the relationship between education and CRF would help improve the efficacy of targeted NCD detection and prevention among high-risk demographic subgroups.

## Abbreviations

95% CI: 95% confidence interval
ANCOVA: Analysis of covariance
BMI: Body mass index
CASMIN: Comparative Analysis of Social Mobility in Industrial Nation classification
CPET: Cardiopulmonary exercise testing
CRF: Cardiorespiratory fitness
CV: Cardiovascular
CVD: Cardiovascular disease
ES: Effect size
HIV/AIDS: Human Immunodeficiency Virus/Acquired Immune Deficiency Syndrome
*I*^2^ statistic: Percentage of variance in the study-specific point estimates that is attributable to true between-study heterogeneity as opposed to sampling variation
LILACS: Latin American and Caribbean Health Sciences
MeSH: Medical subject heading
METS: Metabolic equivalent tasks
NCD: Non-communicable disease
NHANES: National Health and Nutrition Examination Survey
OR: Odds ratio
PA: Physical activity
PAHO: Pan American Health Organization
PRISMA: Preferred Reporting Items for Systematic Review and Meta-Analysis
PROSPERO: International Prospective Register of Systematic Reviews
PWC_170_: Physical working capacity at a heart rate of 170 beats per minute
PWC_75%_: Physical working capacity at 75% of the predicted maximal heart rate
ScIELO: Scientific Electronic Library Online
SES: Socioeconomic status
SHIP: Study of Health in Pomerania
TB: Tuberculosis
VO_2__max_: Maximal oxygen consumption
VO_2__peak_: Peak oxygen consumption
WC: Waist circumference
WHO: World Health Organization
WL_130_: Work load 130, exercise test duration to heart rate of 130 beats per minute
